# New data on distribution, biology, and ecology of longhorn beetles from the area of west Tajikistan (Coleoptera, Cerambycidae)

**DOI:** 10.3897/zookeys.606.9190

**Published:** 2016-07-21

**Authors:** Abdysalom Kh. Kadyrov, Lech Karpiński, Wojciech T. Szczepański, Artur Taszakowski, Marcin Walczak

**Affiliations:** 1 Tajik National University, Rudaki 17, 734025 Dushanbe, Tajikistan; 2 Department of Zoology, Faculty of Biology and Environmental Protection, University of Silesia, Bankowa 9, 40-007 Katowice, Poland

**Keywords:** Central Asia, endemic species, faunistics, invasive species, new records, zoogeography

## Abstract

New data on distribution, biology, and ecology of some little-known cerambycid species, collected in the western part of Tajikistan, are presented. *Arhopalus rusticus* (Linnaeus, 1758) is recorded in Tajikistan for the first time. New localities of species considered pests or invasive species such as *Aeolesthes sarta* (Solsky, 1871) and *Xylotrechus stebbingi* Gahan, 1906 are also given. The list of the taxa collected by the first author during many years of field research in Tajikistan as well as photographs of poorly known species from his collection, including some endemics, are additionally provided. Furthermore, high quality photographs of some extremely rare species that were collected during our expedition, i.e., *Turkaromia gromenkoi* Danilevsky, 2000 and *Ropalopus nadari* Pic, 1894, with images of their habitats or feeding grounds are also presented for the first time.

## Introduction

The longhorn beetle family (Cerambycidae) is one of the most species-rich groups of beetles (Coleoptera) with approximately 35,000 described species (Švácha and Lawrence 2014). The cerambycid fauna of Tajikistan is represented by only 58 species; however, many of them are endemic to the Central Asia region, including those that only occur on the territory of this country (Löbl and Smetana 2010, Danilevsky 2016).

Tajikistan is located almost entirely within the Pamir-Alay Mountain range although forests only cover 2.6% of the area of the country. That places this country on one of the last positions regarding woodiness among the republics of the former Soviet Union. Some relict species such as *Juglans regia*, *Acer turkestanica*, and *Acer reggeli* used to occur in Tajikistan in the past but they are totally extinct as a result of intensive cutting and burning of forests (Rahmonov et al. 2003). Due to a low level of afforestation, cerambycid fauna of Tajikistan is characteristic for the countries of the Central Asia. Species of the subfamily Prioninae which larvae develop in soil feeding on plant roots, as well as many representatives of the tribes of Agapanthiini and Phytoeciini (Lamiinae), whose development takes place in stems and roots of herbaceous plants, dominate in fauna of Tajikistan.

The state of the knowledge of the longhorn fauna of Tajikistan (particularly, of the region of the Pamir Mountains) as well as information about the biology and ecology of some species that are distributed in the region is very poor. Therefore, the present study aims to supplement the knowledge in this field.

## Study area and methods

Tajikistan is a relatively small intra-continental country situated at the boundary of the subtropical and temperate climatic zones. It is located in the mountain desert zone of the Eurasian continent in the southern part of Central Asia, where ecosystems such as deserts, steppes, savannoides, conifer forests, mixed mountain forests, high-mountain deserts, and glaciers are widely represented. The changeable mountain climatic conditions and hard historical natural processes have promoted the formation of a unique biological diversity in Tajikistan, which counts many relict and endemic species (Safarov 2003).

**Map 1.**
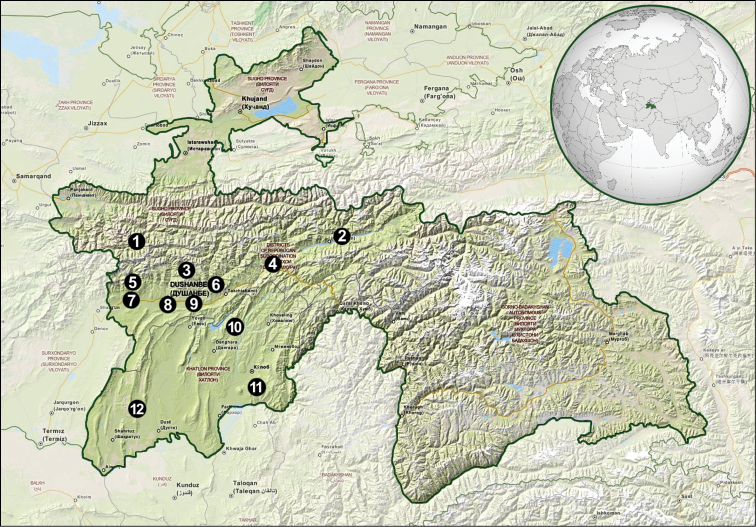
Research plots in the western part of Tajikistan: **1** Iskanderkul **2** Tojikobod **3** Takob **4** Garm **5** Karatag **6** Romit **7** Shahrinav **8** Dushanbe **9** Arykboshi **10** Kangurt **11** Sarichashma **12** Garavuti (OpenStreetMap contributors).

The entomological expedition, which consisted of four scientists from the Department of Zoology, University of Silesia (Poland), took place at the turn of June and July 2014. During the research, several sampling trips were carried out to various locations in the western part of Tajikistan (Map 1). The most extensive studies were conducted within a radius of 60 km from the capital city, Dushanbe, and in the south-western part of the country along the Afghan border. The research were carried out in several research plots in the villages of Arykboshi, Dushanbe, Ganchi, Garavuti, Gharm, Iskanderkul, Kangurt, Karatag, Kolkhozabad, Kulob, Nurobod, Qurghonteppa, Romit, Sarband, Sarichashma, Shahrinav, Shurroabad, Takob, Tojikobod, and Vose.

Tajikistan has a wide variety of habitats that range from gravel deserts in the south through green mountain valleys in the central part of the country to the high mountains with glaciers in the north and east. The area studied includes several different nature ecosystems such as alpine meadows, mesophilic shrubs, various shrub steppes, broad-leaf forests, light forests, and tugay as well as agroecosystems such as gardens, orchards, fields, and pastures.

The most effective, standard methods for collecting beetles such as attracting to artificial light sources, shaking down into an entomological umbrella, sweep netting, and rearing of inhabited material were used during the field research. The beetles were studied using an Optek SZM7045-J4L microscope at 7-90× magnifications. Photographs of the cerambycids in nature, their host plants and habitats were taken with Canon EOS 550D, Canon EOS 600D, and Olympus XZ-1 cameras. Produced images were stacked, aligned, and combined using ZERENE STACKER software (www.zerenesystems.com). Geographical coordinates were read off and recorded using Garmin Oregon 550T 3-Inch Handheld GPS Navigator. For each specimen collected, the exact location (including the GPS coordinates), altitude, date, and the names of the collectors are given. Additionally, information on the general distribution and biology of the species are provided. Some general data that had been collected by the first author during long-term field research were also used.

The following abbreviations are used in the text:

AT
 Artur Taszakowski 


LK
 Lech Karpiński 


MW
 Marcin Walczak 


WTS
 Wojciech T. Szczepański 


The nomenclature was adopted from the Catalogue of Palaearctic Coleoptera (Löbl and Smetana 2010) with further remarks (Danilevsky 2016).

The specimens are preserved in the entomological collection of the Department of Natural History of the Upper Silesian Museum in Bytom and in authors’ collections.

## Results

During the one-month expedition, a total number of 12 species (approximately 20% of the Tajik cerambycid fauna) belonging to three subfamilies (Prioninae 1 sp., Cerambycinae 10 spp., and Lamiinae 1 sp.) were recorded. The list of recorded taxa along with the new localities, general characteristics, and remarks on the species biology and ecology follow.

### Prioninae Latreille, 1802

#### Psilotarsus turkestanicus

Taxon classificationAnimaliaColeopteraCerambycidae

(Semenov, 1888)

Figs 1A, B , 3A

##### Material examined.

Khatlon Region, Sarichashma env. [Саричашма], a semi-ruderal plant community (37°45'N, 69°47'E), 1231 m, 25 VI 2014, 4♂♂, 2♀♀, leg. WTS; 2♂♂, leg. AT; 3♂♂, 1♀, leg. LK; 2♂♂, 1♀, leg. MW.

Although the species is distributed also in Turkmenistan and Tajikistan, most of the known specimens were collected in the Samarkand region in Uzbekistan. In Tajikistan, it was only observed in the north-western part of the country as far as the southern slope of the Gissar Mountain ridge (Danilevsky 2010). The locality in Sarichashma, which is presented for the first time, extends the species range approximately 200 km to the south-east and is one of the first in the country.

Adults are active from early May to late July. In the hilly area of the Katagurgan environs (Uzbekistan), Danilevsky (2014) observed the mass flight of numerous males in the middle of a hot day (between 11 am and 4 pm) on 12 June 1992. This huge number of males was attracted by females standing motionlessly on the ground. Evidently, the daily activity of this species appears to be a unique behaviour among the representatives of the subfamily Prioninae, which is probably correlated with the small size of the eyes in this species in both males and females (Danilevsky 2014).

The larvae develop in soil, where they probably feed on roots of various plants. According to Danilevsky (2014), the larva found in Uzbekistan feeding on roots of *Taraxacum kok*-*saghyz* and described as *Psilotarsus turkestanicus* by Švácha and Danilevsky (1987) certainly belongs to another species common in that region: *Psilotarsus hirticollis hirticollis* Motschulsky, 1860. Therefore, the larval stage of *Psilotarsus turkestanicus* has not been described yet.

According to Danilevsky (2010), species of the genus *Psilotarsus* Motschulsky, 1860 are often characterised by a very high degree of individual variability. This was also confirmed by us during examining of the collected specimens, which differed, inter alia, in details of elytra, punctuation of the pronotum, shape of the scutellum and size of the spikes on the pronotum.

In the Sarichashma environs, the specimens were collected in a semi-ruderal plant community (Fig. 3B, C) characterised by a variety of plants, including single trees. We observed flights of single males at about noon, although most of the specimens moved on the ground where they were fighting with each other and seeking females. Flying specimens sometimes became the prey of the European roller *Coracias garrulous*, which is a very common species in some regions of Tajikistan. The beetles emit characteristic, audible sounds that make it possible to detect their presence. We noticed many circular exit holes in the ground, which probably belong to this species. It is also noteworthy that the culmination of the occurrence of this species probably took place shortly before or during our first visit to this plot on 25 June. However, when we came back to this location on 3 July, we did not find even a single specimen.

Because this site is located directly on the Tajik-Afghan border, it can be expected that this species will also occur in Afghanistan. On the other hand, the bordering Panj River may form a natural migration barrier, particularly for the females, which are probably flightless. It is noteworthy that two other related Prionini species, *Miniprionus pavlovskii* (Semenov, 1935) and *Pogonarthron semenovianum* (Plavilstshikov, 1936), were also recorded in the immediate vicinity of the plot mentioned above (Danilevsky 1999, Lorenc unpublished data).

The authors feel compelled to state that this plot is located in a strongly guarded zone just a few kilometres from the Afghan border. No foreigners are allowed to enter this area without the proper permits. Staying in this zone (especially at night) can have serious consequences from both the Tajik authorities and Taliban fighters from Afghanistan due to the large drug route in the region.

**Figure 1.**
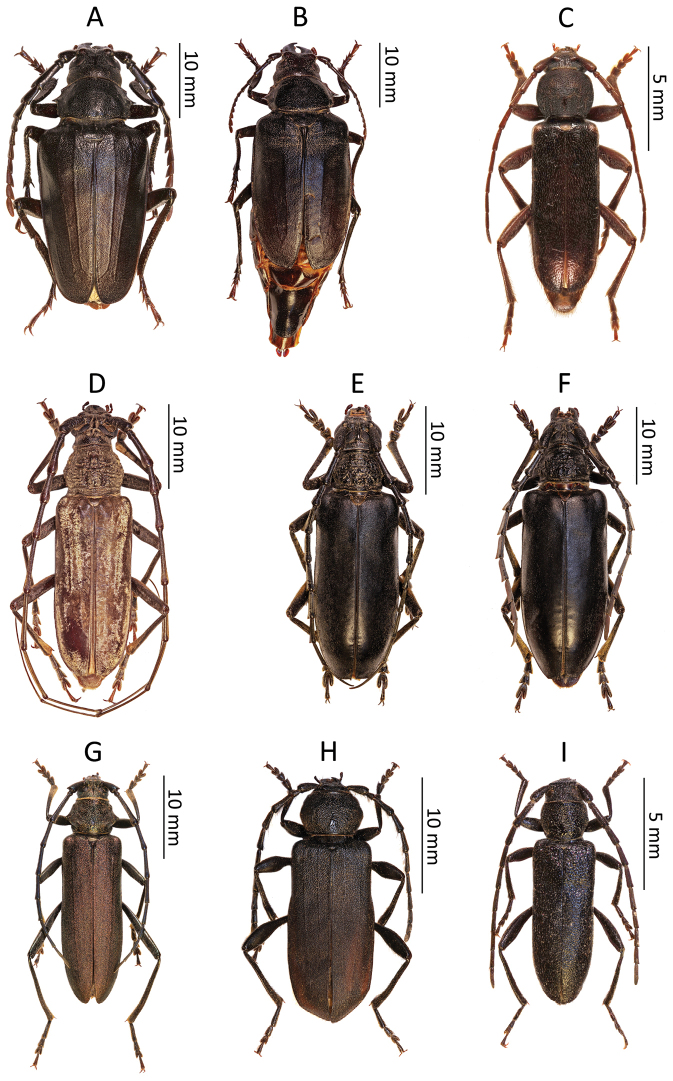
Photos of longhorn beetles specimens collected during the expedition to Tajikistan in 2014: **A** *Psilotarsus turkestanicus* (male) **B** *Psilotarsus turkestanicus* (female) **C** *Trichoferus campestris* **D** *Aeolesthes sarta* **E** *Neoplocaederus scapularis* (male) **F** *Neoplocaederus scapularis* (female) **G** *Turkaromia gromenkoi* **H** *Ropalopus nadari* **I** *Turanium pilosum*.

**Figure 2.**
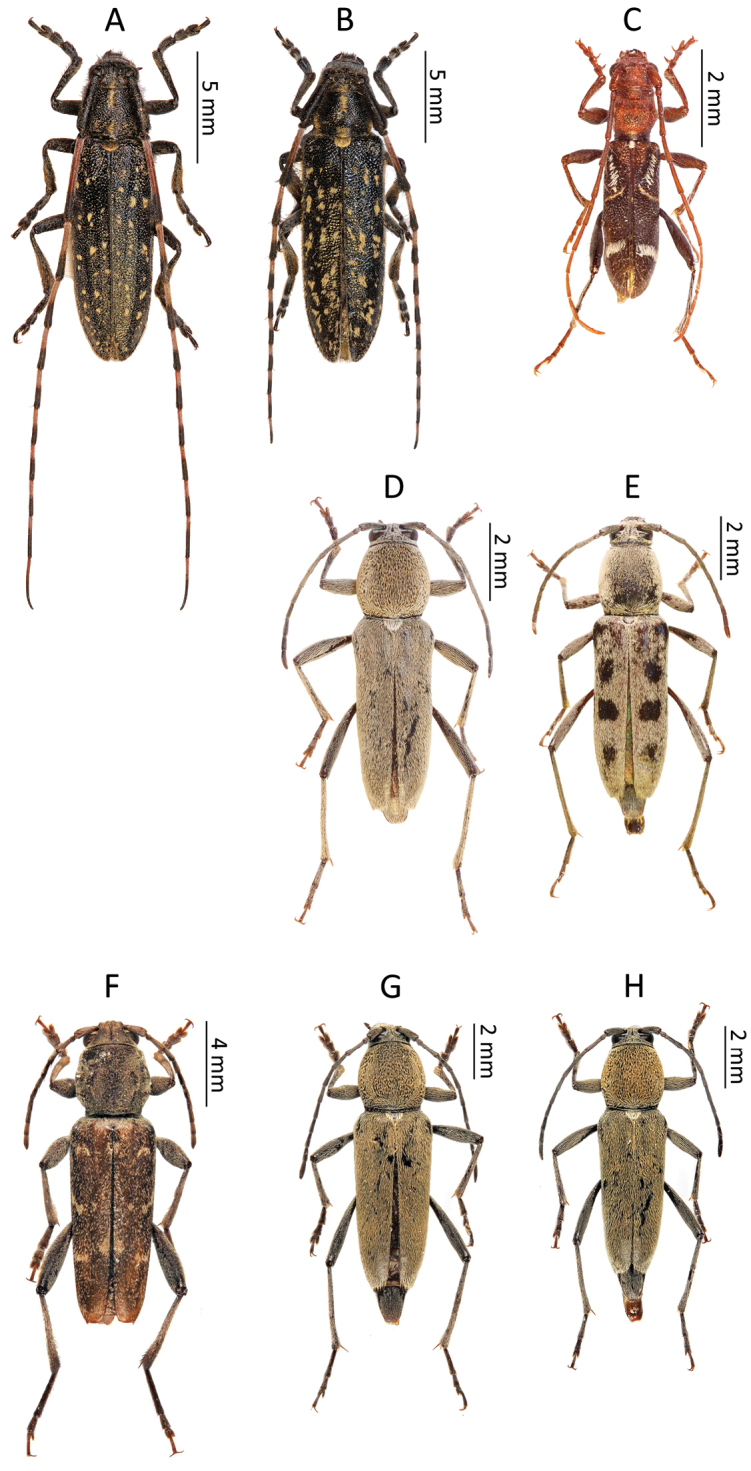
Photos of longhorn beetles specimens collected during the expedition to Tajikistan in 2014: **A** *Agapanthia soror* (male) **B** *Agapanthia soror* (female) **C** *Cleroclytus banghaasi* **D** *Chlorophorus faldermanni* (male) **E** *Chlorophorus faldermanni* (female) **F** *Xylotrechus stebbingi*, **G** *Chlorophorus elaeagni* (male) **H** *Chlorophorus elaeagni* (female).

**Figure 3.**
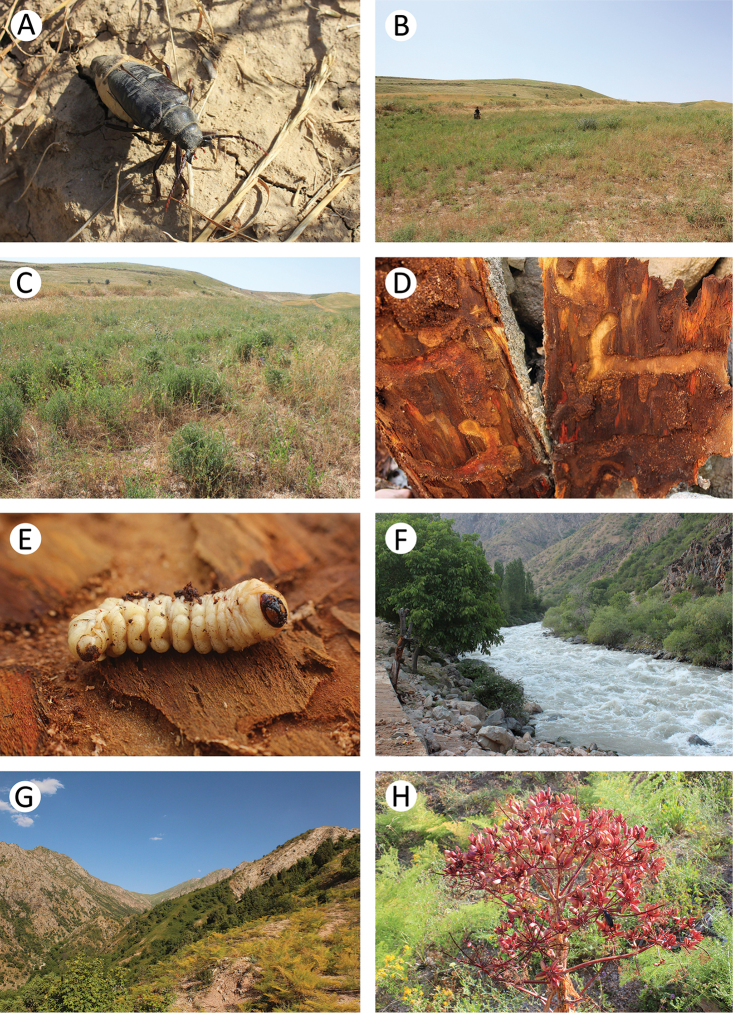
Field photos of imagines in nature, their habitats and larval feeding grounds of several Tajik cerambycid species: **A** female of *Psilotarsus turkestanicus* before laying of eggs **B** general view of the location of *Psilotarsus turkestanicus* **C** detailed view of a semi-ruderal plant community, the habitat of *Psilotarsus turkestanicus* **D** larval feeding grounds of *Aeolesthes sarta* **E** one of the last larval instars of *Aeolesthes sarta* **F** riverside woodlands with dying trees, the habitat of *Aeolesthes sarta* **G** mountain meadow overgrown by *Prangos* and *Ferula*, the habitat of *Agapanthia soror* and *Neoplocaederus scapularis* **H** *Neoplocaederus scapularis* on an overblown inflorescence of *Ferula*.

### Cerambycinae Latreille, 1802

#### Trichoferus campestris

Taxon classificationAnimaliaColeopteraCerambycidae

(Faldermann, 1835)

Fig. 1C

##### Material examined.

Dushanbe env. [Душанбе], an orchard, on *Salix* sp. (38°33‘N, 68°54‘E), 920 m, 29 VI 2014, 1♂, 1♀, leg. MW.

Region of Republican Subordination, Arykboshi [Aрыкбошй], a suburban area, at light (38°34'N, 69°04'E), 906 m, 28 VI 2014, 1♂, leg. LK.

Region of Republican Subordination, Tojikobod (Точикобод), an orchard, at light (39°05'N, 70°51'E), 2223 m, 13 VII 2014, 1♀, leg. LK.

*Trichoferus campestris* is considered an invasive species, which has rapidly increased its range in recent years. Its presence in Europe has recently been confirmed inter alia in Romania (2003) (Dascălu and Serafim 2011), the Czech Republic (2006), Slovakia (2007) (Sabol 2009), and Poland (2009) (Kruszelnicki 2010). However, the species appears to be native to China, Japan, the Korean peninsula, Mongolia, Russia, and Central Asia, including Tajikistan (Grebennikov et al. 2010, Dascalu et al. 2013). The larvae are polyphagous on both deciduous and coniferous trees, although the species seems to prefer light deciduous forests and especially orchards. The adults are active at night from June to August and are often attracted to artificial sources of light (Kadyrov 2007).

#### Aeolesthes (Aeolesthes) sarta

Taxon classificationAnimaliaColeopteraCerambycidae

(Solsky, 1871)

Fig. 1D

##### Material examined.

Dushanbe [Душанбе], city center, at light (38°34'N, 68°44'E), 871 m, 7 VII 2014, 1♂, leg. WTS.

Region of Republican Subordination, Romit [Ромит], river valley, at light (38°46'N, 69°16'E), 1283 m, 26 VI 2014, 1♂, 2♀♀, leg. LK.

Region of Republican Subordination, Karatag [Каратаг], at light, (38°41'N, 68°22'E), 1108 m, 30 VI 2014, 1♀, leg. LK.

The city longhorn beetle *Aeolesthes sarta* is a species widely distributed throughout the Palaearctic and the Oriental region. It is believed that it originated in Pakistan and Western India from which it spread to Afghanistan, Iran, and to Central Asia (Orlinskii et al. 1991).

The species is polyphagous with a wide range of host plants and it primarily attacks tree trunks. For this reason, the species is considered a serious pest in the countries in which it occurs. In many cities of Central Asia, poplars and willows have been destroyed as well as plane trees, acacias, and ashes in Dushanbe (Kadyrov 2007). It takes two years for the larvae to develop. The adults overwinter in a pupal cell and emerge in the following spring. The flight period of adults begins in the second part of April and lasts more or less until mid-July. Imagines are generally active in the evening and night and very often are attracted to sources of light (Kadyrov 2007).

It was observed that *Aeolesthes sarta* attacks both maximally exposed and shaded trees. The larval feeding grounds (Fig. 3D) of this species were additionally found in two other locations: Garavuti env. [37°35'N, 68°31'E] and Shahrinav env. [38°36’N, 68°19'E]. In above-mentioned Romit area, in addition to the imagines that were collected, about 20 larvae of *Aeolesthes sarta* (Fig. 3E) were also found on a dead trunk of *Prunus* sp. (Fig. 3F).

#### Neoplocaederus scapularis

Taxon classificationAnimaliaColeopteraCerambycidae

(Fischer von Waldheim, 1821)

Fig. 1E, F

##### Material examined.

Region of Republican Subordination, Romit [Ромит], a river valley, at light (38°46'N, 69°16'E), 1283 m, 26 VI 2014, 1♂, leg. WTS; 1♂, 2♀♀, leg. LK.

Region of Republican Subordination, Takob [Taкoб], an alpine meadow, on *Ferula* sp., (38°49'N, 68°56'E), 1850–1900 m, 9 VII 2014, 1♂, leg. WTS; 3♀♀, leg. LK.

It is distributed in several countries of Central Asia, Iran, Afghanistan, and western China (Kadyrov 2007, Danilevsky 2016). It is a common species in Tajikistan and occurs everywhere its host plant ferule (*Ferula* spp.) grows (Kadyrov 2007). In Tajikistan, the genus *Ferula* consists of nearly 40 species (Safarov 2003). According to Plavilstshikov (1940), larvae of *Neoplocaederus scapularis* may also develop in species of the genus *Scorodosma*. The larvae primarily feed on the rhizomes and roots of these plants and their development usually takes a year, sometimes even two (Plavilstshikov 1940). Pupation takes place in calcareous cocoons in the soil (Švácha and Danilevsky 1988). The adults appear and feed on the flowers or stems of the host plants from the end of April to July, depending on the local altitude (Kadyrov 2007).

The beetles are probably active in the evening and at night. We only observed adults on the ferule (Fig. 3G, H) in the early morning, due to the fact that the beetles still had not managed to hide after the night. Moreover, during the research, imagines were often attracted to an artificial light source.

#### Turkaromia gromenkoi

Taxon classificationAnimaliaColeopteraCerambycidae

Danilevsky, 2000

Fig. 1G

##### Material examined.

Sughd Region, Iskanderkul [Искандарkӯл], bushes near a river valley (39°05'N, 68°24'E), 2300 m, 18 VII 2014, 1♀, leg. AT.

The genus *Turkaromia* Danilevsky, 1993 was quite recently separated by Danilevsky (1993) and includes two species, *Turkaromia pruinosa* (Reitter, 1903) and *Turkaromia gromenkoi*, which are distributed in the region of Central Asia. According to Danilevsky (2000), *Turkaromia gromenkoi* is distributed in the western part of the Gissar Mountain ridge in Uzbekistan and Tajikistan. The species was described from four specimens: one male and two females from Kaltakol (Uzbekistan) and one female from Iskanderkul (Tajikistan). All specimens were observed in July. The biology and ecology of species as well as the stages of the larvae and pupae are unknown.

**Figure 4.**
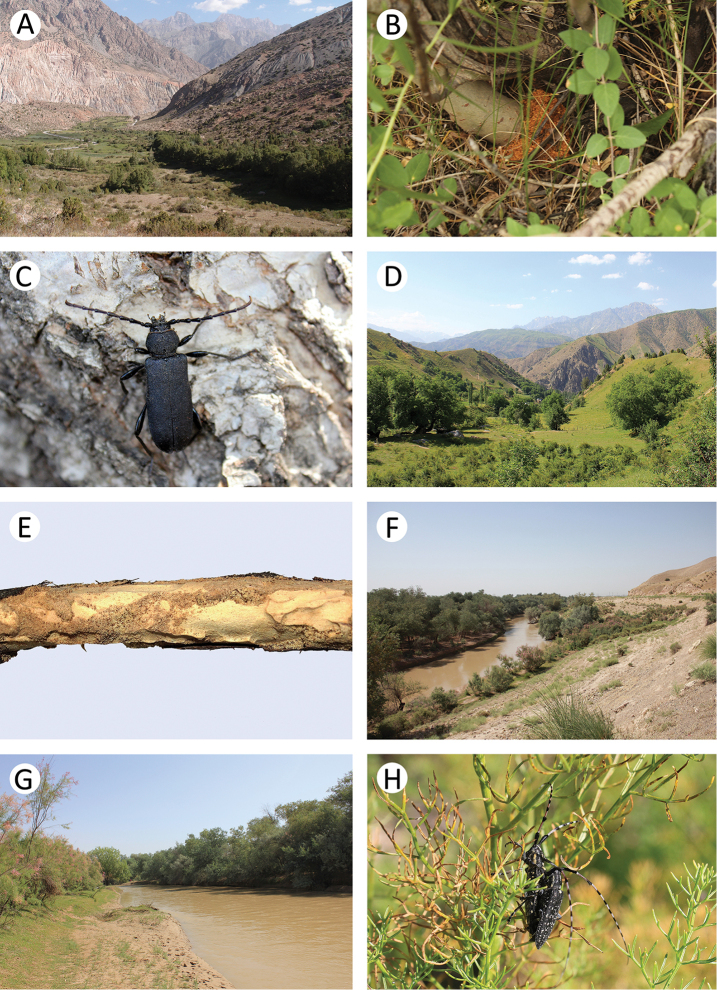
Field photos of imagines in nature, their habitats and larval feeding grounds of several Tajik cerambycid species: **A** birch and willow bushes near a river valley, the habitat of *Turkaromia gromenkoi* **B** sawdust-like waste on the outside of the trunk of a middle-aged willow, the probable result of the larval feeding of *Turkaromia gromenkoi* **C** female of *Ropalopus nadari* on the bark of *Malus sieversii* **D** walnut and apple trees in a mountain valley, the habitat of *Turanium pilosum* and *Ropalopus nadari* **E** larval feeding grounds of *Turanium pilosum* **F** tugay in the Vakhsh River valley, the habitat of *Chlorophorus elaeagni* and *Chlorophorus faldermanni* **G** tugay with blossoming *Tamarix* in the Vakhsh River valley **H** male and female of *Agapanthia soror* in copula on *Prangos*.

In the environs of the Iskanderkul Lake, we observed one female on a flower (Apiaceae) in a biotope near a river valley that had been overgrown by willows (*Salix* spp.) and shan birches *Betula tianschanica* (Fig. 4A). The larvae probably develop in the living wood of willows similar to the related species *Turkaromia pruinosa*. In the immediate vicinity of the area where the beetle was collected, we found sawdust-like waste on the outside of the trunk of a middle-aged willow (Fig. 4B), which was probably the result of the larval feeding of *Turkaromia gromenkoi*.

It is noteworthy that only one specimen was found despite a few hours of examining the plot using various methods. The presence of only a single female may indicate the end of the period of the occurrence of this species. It appears that *Turkaromia gromenkoi* is endemic to the Gissar Mountains.

#### Ropalopus (Ropalopus) nadari

Taxon classificationAnimaliaColeopteraCerambycidae

(Pic, 1894)

Figs 1H , 4C

##### Material examined.

Region of Republican Subordination, Takob [Taкoб], an alpine meadow, on *Malus sieversii* (38°49'N, 68°56'E), 1850 m, 9 VII 2014, 1♀, leg. WTS.

This species occurs in Kyrgyzstan, Tajikistan, and Uzbekistan (Danilevsky 2016) and is endemic to the region of western Tian Shan Range (Kadyrov 2007). *Ropalopus nadari* is a polyphagous species, which usually inhabits growing wild fruit trees in the upper zone of deciduous forests (Fig. 4D). This species most frequently inhabits trunks and boughs of the wild apple tree *Malus sieversii*. Its larval development usually takes two years (Plavilstshikov 1940, Kadyrov 2007). Adults are found from June to July. After they emerge, the imagines are unwilling to fly and generally stay on the host plant and only visit flowers of *Ferula* spp. and *Prangos* spp. occasionally (Kadyrov 2007).

Only a single female was observed despite a 24-hour monitoring of the trees on the plot. This may either be related to the end of the period of the occurrence of the species or its hidden life in the treetops.

#### Turanium (Turanium) pilosum

Taxon classificationAnimaliaColeopteraCerambycidae

(Reitter, 1891)

Fig. 1I

##### Material examined.

Region of Republican Subordination, Takob [Taкoб], on a tree branch fence, (38°49'N, 68°56'E), 1850 m, 9 VII 2014, 3♂♂, 1♀, leg. WTS; 2♂♂, 1♀, leg. LK; 1♂, leg. MW; (10 II 2015, 2♀♀, ex cult. *Malus sieversii*), leg. WTS; (7–21 XII 2014, 2♀♀, ex cult. *Malus sieversii*), leg. LK; (11–21 I 2015, 2♂♂, ex cult. *Malus sieversii*), leg. MW.

This species is distributed in the countries of Central Asia and the Xinjiang region of China (Danilevsky 2016). It inhabits the upper zone of deciduous forests and, less frequently, valleys. Although *Turanium pilosum* is polyphagous on deciduous trees (Kadyrov 2007), the larvae can also feed on conifers (Danilevsky 2001a). The species inhabits dry twigs and stems (Fig. 4E). Its development usually takes two years with pupation in spring. The adults fly from April to August (Danilevsky 2001a, Kadyrov 2007).

Mating of this species seems to start at the end of June. We observed adults flying into wooden components and actively moving on trunks of the wild apple tree *Malus sieversii*, where they were also mating. *Turanium pilosum* was recorded sympatrically with *Ropalopus nadari* in the same habitat (Fig. 4D).

#### Xylotrechus (Xylotrechus) stebbingi

Taxon classificationAnimaliaColeopteraCerambycidae

Gahan, 1906

Fig. 2F

##### Material examined.

Region of Republican Subordination, Arykboshi [Aрыкбошй], on the wood piles of *Juglans* sp. (38°34'N, 69°04'E), 906 m, 28 VI 2014, 2♂♂, 3♀♀, leg. WTS; 2 VII 2014, 3♂♂, 2♀♀, leg. LK.; 28 VI 2014, at light 1♂ leg. LK.

Khatlon Region, Чаврок, N of Kangurt, at light (38°18'N, 69°32'E), 1217 m, 5 VII 2014, 1♂, 1♀, leg. LK.

This is a widely distributed species, whose origin is not clear (Cocquempot and Lindelöw 2010). It probably originally came from the region of northern India (Himalayas, Tibet). In recent years, this invasive species has spread to and become acclimated in the Middle East, the Mediterranean region (Sama 2002), and possibly in Central Asia. In Europe, it was recorded for the first time in Italy in 1990 (Dioli and Viganò 1990). The larvae are polyphagous on broad-leaved trees. Its life cycle lasts two years. Adults are usually encountered between May and November (Sama 2002, Ali et al. 2015).

It was observed that this species appears to be strongly synanthropic in Tajikistan: adults were found in various anthropogenic environments such as backyards and orchards, where they willingly flew to artificial light sources.

#### Cleroclytus (Obliqueclytus) banghaasi

Taxon classificationAnimaliaColeopteraCerambycidae

(Reitter, 1895)

Fig. 2C

##### Material examined.

Region of Republican Subordination, 30 km SW of Garm [Ғарм], Yakhoh env. (38°51'N, 70°01'E), ca 1300 m, 11 VII 2014 (8 XII 2014, 1♂; 21 XI 2014, 1♂; 28 XII 2014, 1♀, ex cult.), leg. LK; (28 XII 2014 – 26 I 2015, 2♂♂, 6♀♀, ex cult.), leg. MW.

This species occurs in Kyrgyzstan and Tajikistan (Danilevsky 2016), where it is widely distributed in both mountain areas and valleys. It is polyphagous on deciduous trees. The larvae feed subcortically on recently dead twigs or branches. Larva overwinters and then it pupates in the wood in the following spring; sometimes the imago overwinters. The adults fly from April to June and feed on flowers, especially those of *Cerasus*, *Prunus*, *Malus*, *Pyrus*, *Rosa*, *Cotoneaster*, *Atraphaxis*, and *Exochorda* (Kadyrov 2007).

#### Chlorophorus (Immaculatus) elaeagni

Taxon classificationAnimaliaColeopteraCerambycidae

Plavilstshikov, 1956

Fig. 2G, H

##### Material examined.

Region of Republican Subordination, Shahrinav env. (Шаxринав), on the flowers of *Tamarix* sp. and on Apiaceae (38°36'N, 68°19'E), 868 m, 2 VII 2014, 1♂, leg. LK; 2 VII 2014, 5♂♂, 2♀♀, leg. MW; 1♀, leg. AT.

Khatlon Region, Garavuti env. [Ғаравӯтӣ], on the flowers of *Tamarix* sp. (37°35'N, 68°31'E), 356 m, 24 VI 2014, 2♂♂, leg. WTS; 2♂♂, leg. MW.

This species is distributed from the Caucasus to Central Asia (Danilevsky 2016) where it mainly inhabits floodplains and riparian forests (tugay) (Fig. 4F, G). The larvae feed on the dead wood of various deciduous trees. They were recorded inter alia on *Elaeagnus*, *Halimodendron*, *Caragana*, and *Robinia*. The life cycle of this species usually takes two years with pupation in spring and early summer. The adults feed on various flowers from April to July (Svácha and Danilevsky 1988, Kaliuzhnaja et al. 2000, Kadyrbekov and Tleppaeva 2004).

#### Chlorophorus (Immaculatus) faldermanni

Taxon classificationAnimaliaColeopteraCerambycidae

(Faldermann, 1837)

Fig. 2D, E

##### Material examined.

Region of Republican Subordination, Shahrinav env. (Шаxринав), on the flowers of *Tamarix* sp. and on Apiaceae (38°36'N, 68°19'E), 868 m, 2 VII 2014, 2♂♂, 1♀, leg. WTS; 1♂, 2♀♀, leg. MW; 1♂, leg. AT.

This species is distributed in the Caucasus, the Far East, Central Asia, and Oriental region (Danilevsky 2016). It occurs in valleys as well as in mountain areas up to 2500 m. Like the previous species, it is rather common in a tugay habitat. It is polyphagous on deciduous trees, mostly on poplars and willows (Kadyrov 2007), although it also inhabits tamarisks *Tamarix*, oleasters *Eleagnus*, elms *Ulmus* and pears *Pirus* (Švácha and Danilevsky 1988, Shapovalov 2012). Furthermore, its larvae also develop in wooden structures, which makes *Chlorophorus faldermanni* one of the most serious pests of timber in Central Asia. Its life cycle lasts one or two years. Its flight period is from May to September (Švácha and Danilevsky 1988, Kadyrov 2007). The adults frequently visit flowers, especially Apiaceae (Kadyrov 2007).

This species was observed sympatrically with *Chlorophorus elaeagni* on blossoming tamarisks (*Tamarix* spp.) (Fig. 4G).

### Lamiinae Latreille, 1825

#### Agapanthia (Stichodera) soror

Taxon classificationAnimaliaColeopteraCerambycidae

Kraatz, 1882

Figs 2A, B , 4H

##### Material examined.

Region of Republican Subordination, Karatag [Каратаг] (38°43'N, 68°22'E), 1108 m, 30 VI 2014, 1♀, leg. MW.

Region of Republican Subordination, Takob [Taкoб], alpine meadow, on *Ferula* sp., (38°49'N, 68°56'E), 1850–1900 m, 8 VII 2014 – 9 VII 2014, 10♂♂, 2♀♀, leg. AT; 22♂♂, 16♀♀, leg. WTS; 16♂♂, 8♀♀, leg. LK; 20♂♂, 17♀♀ leg. MW.

Region of Republican Subordination, Tojikobod (Точикобод), alpine meadow (39°05'N, 70°51'E), 2223 m, 13 VII 2014, 2♂♂, leg. AT.

This species occurs in Tajikistan, Uzbekistan, Kyrgyzstan, and Kazakhstan (Danilevsky 2016). It is common in alpine meadow at altitudes of between 1000 and 3000 m (Fig. 3G). The larvae feed on the stems and rhizomes of *Prangos* spp. The adults feed on host plants from May until the end of July, although they were also observed on mallows *Malva* spp. The imagines usually stay on the stems of host plants, where they copulate and supplementary feed during summer. After that, the females lay eggs in the incisions in the stems. A female usually lays only one egg on each stem. In 8 to 12 days, the larva hatches and bites into the core of the stem where it moves towards the root. The larva forms a pupal cell and overwinters in the lower part of stem or the upper part of root. Pupation occurs in the spring (Kadyrov 2007).

A massive mating of this species was observed in the Takob environs between 8 and 10 July. The beetles performed characteristic slow flights during the day. It is noteworthy that no more species of the genera *Agapanthia* or *Phytoecia* were caught during the entire expedition, despite the very frequent use of the sweep-netting method in appropriate habitats (e.g. alpine meadows) in various parts of the country. This species seems to occur much later or longer than the other related species.

### Checklist of the Cerambycidae of Tajikistan

The followed list is based on Danilevsky (2016). Species collected by the first author over many years of research are marked with an asterisk (*). Endemic taxa are marked with a letter (E).

**Prioninae** Latreille, 1802

**Figure 5.**
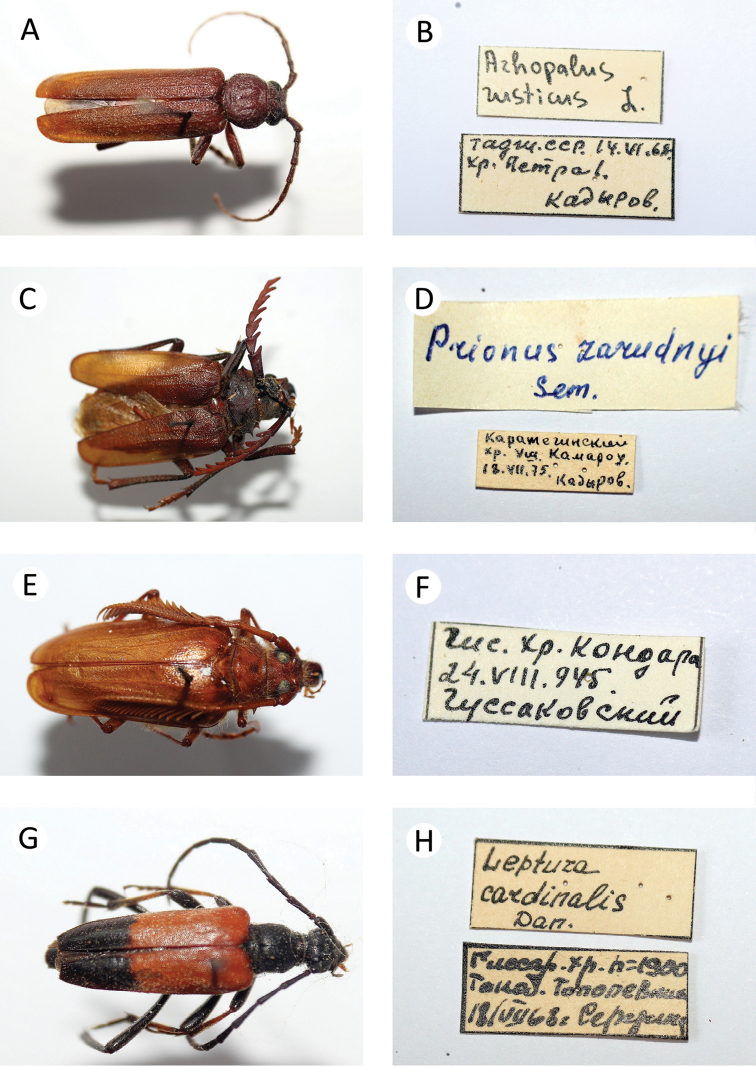
Several longhorn beetles specimens from the collection of the first author: **A** *Arhopalus rusticus rusticus* **B** label of *Arhopalus rusticus rusticus* – first record from Tajikistan **C** *Mesoprionus zarudnii* **D** label of *Mesoprionus zarudnii* **E** *Pogonarthron bedeli* **F** label of *Pogonarthron bedeli* **G** *Stictoleptura cardinalis* **H** label of *Stictoleptura cardinalis*.

1. *Mesoprionus angustatus* (Jakovlev, 1887) *

2. *Mesoprionus zarudnii* (Semenov, 1933) * E (Fig. 5C, D)

3. *Psilotarsus hirticollis hirticollis* Motschulsky, 1860

4. *Psilotarsus turkestanicus* (Semenov, 1888)

5. Pogonarthron (Multicladum) semenovianum (Plavilstshikov, 1936) E

6. Pogonarthron (Pogonarthron) bedeli (Semenov, 1900) * E (Fig. 5E, F)

7. Pogonarthron (Pogonarthron) petrovi ivanovae Pak & Skrylnik, 2014 E

8. Pogonarthron (Pogonarthron) petrovi petrovi Danilevsky, 2004 E

9. *Miniprionus pavlovskii* (Semenov, 1935) E

10. *Microarthron komaroffi* (Dohrn, 1885)

**Lepturinae** Latreille, 1802

**Figure 6.**
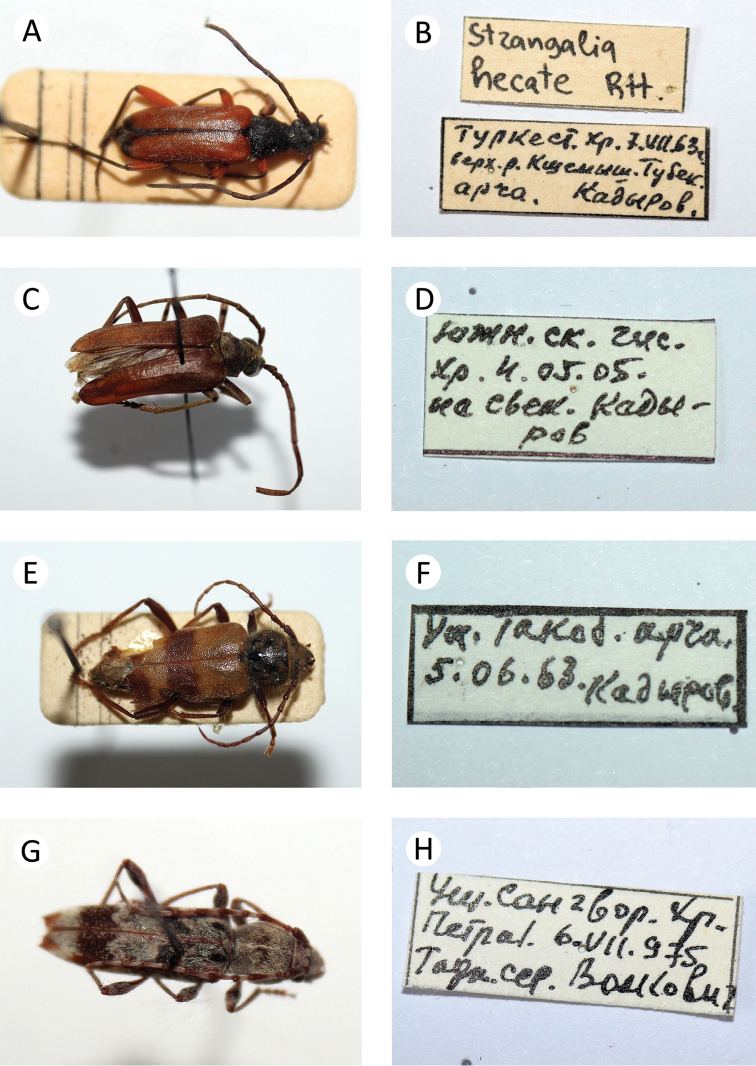
Several longhorn beetles specimens from the collection of the first author: **A** *Xenoleptura hecate* **B** label of *Xenoleptura hecate* **C** *Apatophysis pavlovskii* **D** label of *Apatophysis pavlovskii* **E** *Semanotus semenovi* **F** label of *Semanotus semenovi* **G** *Anaglyptus bicallosus* **H** label of *Anaglyptus bicallosus*.

11. Stictoleptura (Stictoleptura) cardinalis (K. Daniel & J. Daniel, 1898) * (Fig. 5G, H)

12. *Xenoleptura hecate* (Reitter, 1896) * (Fig. 6A, B)

**Spondylidinae** Audinet-Serville, 1832

13. *Arhopalus rusticus rusticus* (Linnaeus, 1758) * (Fig. 5A, B) – first record for Tajikistan

**Apatophyseinae** Lacordaire, 1869

14. Apatophysis (Apatophysis) pavlovskii Plavilstshikov, 1954 * (Fig. 6C, D)

15. Apatophysis (Apatophysis) komarowi Semenov, 1889

**Cerambycinae** Latreille, 1802

**Figure 7.**
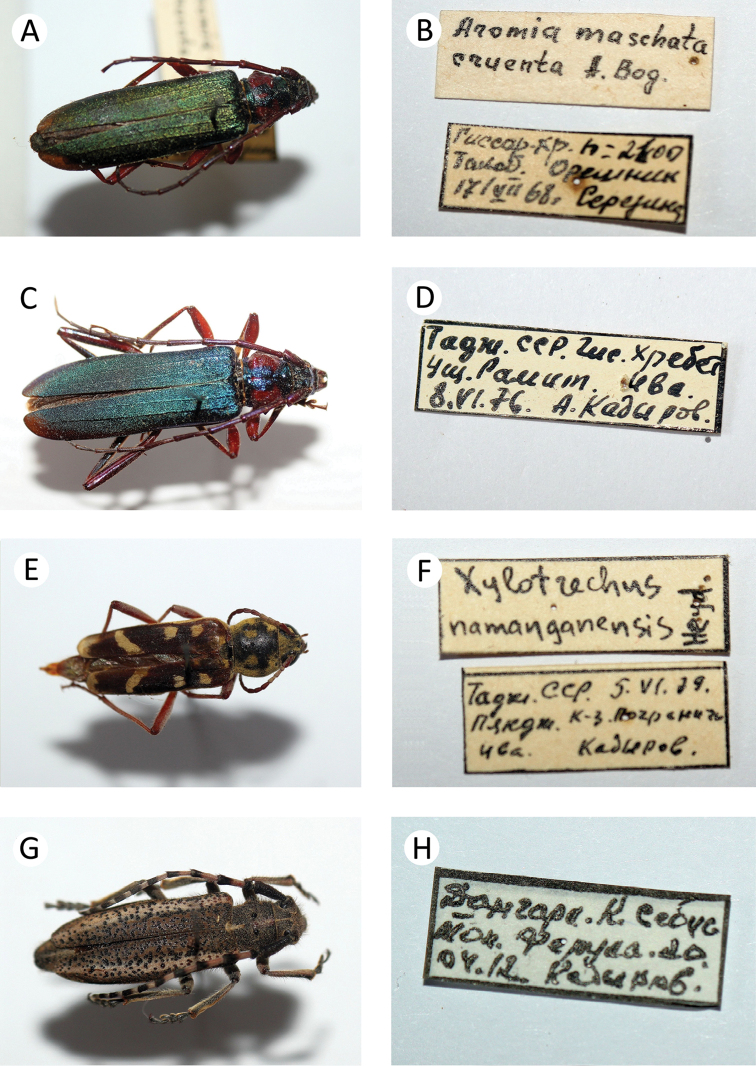
Several longhorn beetles specimens from the collection of the first author: **A** *Aromia moschata cruenta* **B** label of *Aromia moschata cruenta* **C** *Aromia moschata cruenta* **D** label of *Aromia moschata cruenta* **E** *Xylotrechus namanganensis* **F** label of *Xylotrechus namanganensis* **G** *Mallosiola regina* **H** label of *Mallosiola regina*.

16. *Trichoferus campestris* (Faldermann, 1835)

17. Aeolesthes (Aeolesthes) sarta (Solsky, 1871) *

18. *Neoplocaederus scapularis* (Fischer von Waldheim, 1821) *

19. *Aromia moschata cruenta* Bogatchev, 1962 * (Fig. 7A–D)

20. *Turkaromia gromenkoi* Danilevsky, 2000

21. Ropalopus (Ropalopus) nadari (Pic, 1894) *

22. Turanium (Turanium) pilosum (Reitter, 1891) *

23. Turanium (Turanium) scabrum (Kraatz, 1882) *

24. *Semanotus semenovi* Okunev, 1933 * (Fig. 6E, F)

25. Cleroclytus (Obliqueclytus) banghaasi (Reitter, 1895) *

26. Cleroclytus (Obliqueclytus) gracilis Jakovlev, 1900 * E

27. Anaglyptus (Anaglyptus) bicallosus (Kraatz, 1882) * (Fig. 6G, H)

28. *Echinocerus floralis* (Pallas, 1773)

29. Chlorophorus (Immaculatus) elaeagni Plavilstshikov, 1956 *

30. Chlorophorus (Immaculatus) faldermanni (Faldermann, 1837) *

31. Chlorophorus (Humeromaculatus) navratili Holzschuh, 1981

32. Xylotrechus (Xylotrechus) stebbingi Gahan, 1906

33. Xylotrechus (Turanoclytus) asellus (Thieme, 1881)

34. Xylotrechus (Turanoclytus) namanganensis (Heyden, 1885) * (Fig. 7E, F)

35. Xylotrechus (Rusticoclytus) rusticus (Linnaeus, 1758)

**Lamiinae** Latreille, 1825

**Figure 8.**
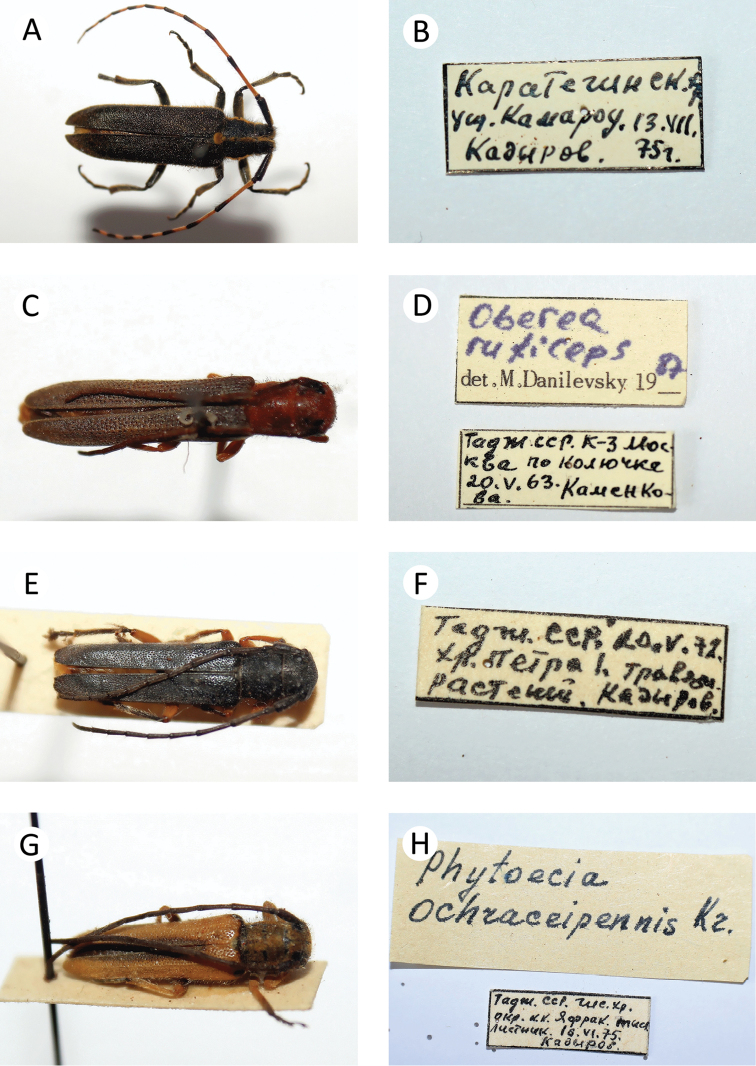
Several longhorn beetles specimens from the collection of the first author: **A** *Agapanthia detrita* **B** label of *Agapanthia detrita* **C** *Oberea ruficeps muchei* **D** label of *Oberea ruficeps muchei* **E** *Phytoecia eylandti* **F** label of *Phytoecia eylandti* **G** *Phytoecia ochraceipennis* **H** label of *Phytoecia ochraceipennis*.

36. Dorcadion (Ciberodorcadion) dokhtouroffi Ganglbauer, 1886

37. Dorcadion (Ciberodorcadion) turkestanicum Kraatz, 1881

38. Saperda (Saperda) similis Laicharting, 1784 *

39. Oberea (Amaurostoma) ruficeps muchei Breuning, 1981 * E (Fig. 7C, D)

40. *Mallosiola regina* Heyden, 1887 * E (Fig. 7G, H)

41. Phytoecia (Pseudocoptosia) cinerascens Kraatz, 1882 *

42. Phytoecia (Pseudocoptosia) eylandti Semenov, 1891 * (Fig. 8E, F)

43. Phytoecia (Pseudocoptosia) kubani Holzschuh, 1991 E

44. Phytoecia (Fulgophytoecia) circumdata Kraatz, 1882 *

45. Phytoecia (Phytoecia) acridula Holzschuh, 1981 *

46. Phytoecia (Phytoecia) caerulea caerulea (Scopoli, 1772)

47. Phytoecia (Phytoecia) pustulata pustulata (Schrank, 1776)

48. Phytoecia (Phytoecia) rufipes rufipes (Olivier, 1795) *

49. Phytoecia (Phytoecia) virgula (Charpentier, 1825) *

50. Phytoecia (Opsilia) bucharica Breuning, 1943

51. Phytoecia (Opsilia) coerulescens Scopoli, 1763 *

52. Phytoecia (Opsilia) varentzowi Semenov, 1896

53. Phytoecia (Blepisanis) nivea Kraatz, 1882

54. Phytoecia (Blepisanis) ochraceipennis Kraatz, 1882 * (Fig. 7G, H)

55. Agapanthia (Epoptes) dahli dahli (C. F. W. Richter, 1820)

56. Agapanthia (Epoptes) detrita Kraatz, 1882 * (Fig. 8A, B)

57. Agapanthia (Epoptes) ustinovi Danilevsky, 2013 E

58. Agapanthia (Stichodera) soror Kraatz, 1882 *

59. Agapanthia (Smaragdula) incerta Plavilstshikov, 1930 *

60. *Agapanthiola leucaspis* (Steven, 1817)

## Discussion

Knowledge about the fauna of Tajikistan, particularly invertebrates, is still poor. Species of beetles that are new to science are increasingly being described, for example, *Kytorhinus kergoati* Delobel & Legalov, 2009 (Chrysomelidae) (Delobel and Legalov 2009), *Trachelanthus lopatini* Korotyaev & Nasreddinov, 2013 (Curculionidae) (Korotyaev and Nasreddinov 2013), *Meloe kulabensis* Shapovalov, 2014 (Meloidae) (Shapovalov 2014), or *Dryops renateae* Greń & Przewoźny, 2016 (Dryopidae) (Greń et al. 2016).

Longhorn beetles arouse great interest among beetle families, and therefore the current state of knowledge on Tajik cerambycids appears to be better than that for other groups. However, the knowledge about the species that occur in this country is still insufficient; thus, some new taxa have been described in recent years: *Turkaromia gromenkoi* (Danilevsky 2000), *Pogonarthron petrovi petrovi* (Danilevsky 2004), *Pogonarthron petrovi ivanovae* (Pak and Skrylnik 2014), and *Agapanthia ustinovi* (Danilevsky 2013).

The distribution of species within the individual subfamilies is characteristic for Central Asia and it is presented as follows: Prioninae (9), Lepturinae (2), Spondylidinae (1), Apatophyseinae (2), Cerambycinae (20), and Lamiinae (25). Species of the subfamily Prioninae, whose larvae develop in soil and feed on plant roots, as well as many representatives of the tribes of Agapanthiini and Phytoeciini (Lamiinae), whose development take place in the stems and roots of herbaceous plants, dominate the fauna of Tajikistan. Representatives of the subfamily Lepturinae are few in number possibly due to the lack of trees and, consequently, small amounts of deadwood, which is quite a normal situation in Central Asia. For example, the total number of Lepturinae species is: 5 (Uzbekistan), 6 (Turkmenistan), 9 (Kyrgyzstan), and 4 (Afghanistan).

Because of the many geographical barriers in Tajikistan, a high level of endemism occurs in this group of beetles. Currently among the 60 taxa, as many as eleven taxa occur exclusively in Tajikistan. The endemics include *Mesoprionus zarudnii*, *Pogonarthron semenovianum* (all known specimens were only collected in Tajikistan, near the Afghan border (Danilevsky 2004), but according to Danilevsky (2016) this species occurs also in Afghanistan), *Pogonarthron bedeli*, *Pogonarthron petrovi petrovi*, *Pogonarthron petrovi ivanovae*, *Miniprionus pavlovskii*, *Cleroclytus gracilis*, *Agapanthia ustinovi*, *Mallosiola regina*, *Oberea ruficeps muchei*, and *Phytoecia kubani*.

Due to its climatic conditions, Tajikistan is a very unique place to collect insects. The flight period of various groups of longhorn beetles is quite diverse. Some species only occur in spring, for example the genus *Phytoecia*, which is represented here by as many as 13 species. On the other hand, some species of the subfamily Prioninae begin to fly in late summer. There are also many nocturnal species that lead very cryptic lifestyles. For these reasons, comprehensive studies that include the entire growing season are needed to obtain a reasonably full and true picture of the composition of the species of longhorn beetles in this country.

Today, the industrial and economic activities of humans are the most important factors that influence ecosystems. Such anthropogenic activities cause important changes in fauna and flora that lead to the simplification of biogeocenosis structures and to decrease in the differences between landscape zones. Some agrotechnical measures, such as ploughing desert regions of a country, deforestation, intensive irrigation, chemical use, and the development of industry in large parts of southeast Central Asia have induced the development of specific fauna in the anthropogenic landscapes. Among the most important forms of human activity are ploughing and managing soils that had not been used earlier, which causes the formation of secondary biocoenosis and agrocoenosis, which in turn leads to disturbances of the ecosystems and, as a result, to the disappearance of many vulnerable species. Planting trees and shrubs in disturbed regions is extremely important due to their role as an ecological corridor (Rahmonov et al. 2003).

Other than the extensive research activities of the first author (e.g. Kadyrov 1989, 2007) and Danilevsky (e.g. 2001a, b), we do not know of any other studies on Cerambycidae in Tajikistan, with the exception of the activity of beetle collectors. In spite of the fact that access to the region of the Pamir Mountains is severely limited and probably requires special permits for entry, it seems to be particularly interesting in the context of research on longhorn beetles. We still do not know enough about the biology of the local endemic species and there are probably some species that are as yet undiscovered, and therefore new expeditions to this region are quite desirable.

## Supplementary Material

XML Treatment for Psilotarsus turkestanicus

XML Treatment for Trichoferus campestris

XML Treatment for Aeolesthes (Aeolesthes) sarta

XML Treatment for Neoplocaederus scapularis

XML Treatment for Turkaromia gromenkoi

XML Treatment for Ropalopus (Ropalopus) nadari

XML Treatment for Turanium (Turanium) pilosum

XML Treatment for Xylotrechus (Xylotrechus) stebbingi

XML Treatment for Cleroclytus (Obliqueclytus) banghaasi

XML Treatment for Chlorophorus (Immaculatus) elaeagni

XML Treatment for Chlorophorus (Immaculatus) faldermanni

XML Treatment for Agapanthia (Stichodera) soror
